# Ronald W. Busuttil, M.D., Ph.D.— TTS 2024 Medawar Prize Laureate

**DOI:** 10.3389/frtra.2024.1530925

**Published:** 2025-01-22

**Authors:** Jerzy W. Kupiec-Weglinski

**Affiliations:** The Dumont-UCLA Transplantation Center, Department of Surgery, Division of Liver and Pancreas Transplantation, David Geffen School of Medicine at UCLA, Los Angeles, CA, United States

**Keywords:** Ronald Busuttil, Medawar Prize, liver transplantation, surgical scholar, ischemia-reperfusion injury

## Abstract

The Transplantation Society (TTS) has been presenting the Medawar Prize at its biennial Congresses since 1990 in recognition of Sir Peter Medawar's seminal contributions to organ transplantation. This prestigious award acknowledges individuals for their outstanding accomplishments in experimental and clinical transplantation. On September 25, 2024, I was honored to introduce Ronald W. Busuttil, M.D., Ph.D., as the 2024 Medawar Prize Laureate during the 30th TTS Congress in Istanbul, Turkey. This article highlights the remarkable achievements and critical milestones in Dr. Busuttil's over 40-year career in organ transplantation, which have profoundly advanced scientific knowledge and clinical practice, embodying the true spirit of this accolade.

Dr. Ronald W. Busuttil (Figure 1), known as “Dr. B” or “Ronnie” among friends, is currently a Distinguished Professor and Executive Chairman Emeritus at the University of California Los Angeles (UCLA) in the Department of Surgery and the founding Chief of the Division of Liver and Pancreas Transplantation at Ronald Reagan Medical Center, Los Angeles, California, USA.

**Figure 1:**
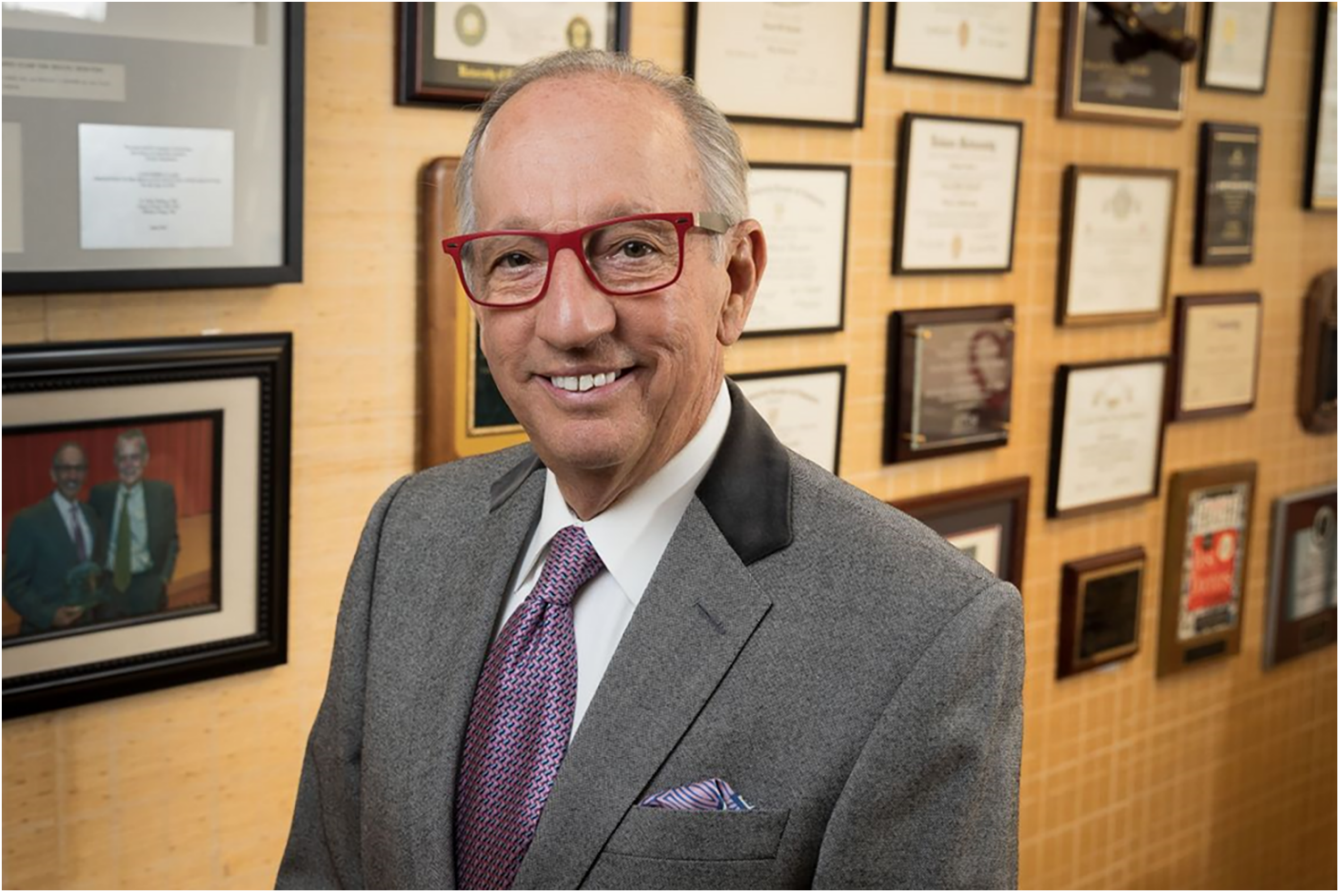
Dr. Ronald W. Busuttil, TTS 2024 Medawar Prize Recipient (Credit: Reed Hutchinson).

The desire to pursue a medical career began in his teenage years, inspired by paternal great-grandfather, who was the private dentist to the king of Egypt. The Busuttil family, with Maltese and Italian roots, resided in Egypt until the Arab-Israeli War in 1948, prompting their relocation to the United States when Ronnie was only four, ultimately settling in Tampa, Florida. He graduated *magna cum laude* from Loyola University in New Orleans and later earned his M.D. and Ph.D. in pharmacology at Tulane University, under the tutelage of future Nobel Laureate, Lou Ignarro. Years later, Dr. Ignarro commended his former student as an exceptionally skilled surgical operator, highlighting his precision during cardiac ischemia experiments on dogs, which were conducted “without a single drop of blood on the floor.”

Dr. Busuttil has dedicated his entire career to UCLA, where he first trained as a vascular surgeon, became faculty, and founded the Liver Transplant Program in 1984, one of the first in the country. For over 36 years, he has served as its Director and Chief Surgeon, making diverse and significant contributions to the field. His outstanding surgical expertise, demonstrated through over 7,200 liver transplants, including more than 1,000 children, at UCLA, UC Irvine, and Cedar Sinai, has critically advanced the care and management of transplant patients globally. The program ranks among the largest in the world, covering a comprehensive range of transplantation and hepato-biliary surgery for adults and children. One of the hallmark areas of Dr. B's clinical innovation addresses the crucial problem in our field, i.e., the donor organ shortage. His pioneering efforts have facilitated the life-saving expansion of the organ pool by refining donor-recipient matching criteria (1), transplantation for malignancies (2), implementing technical modifications of extended criteria donor allografts (1), deceased split donor livers (3), and living donations (4). An exceptional clinician and educator at the bedside, he was instrumental in formulating key concepts that enhanced the care and survival of liver transplant patients.

Dr. Busuttil recalls the day he performed his first liver transplant at UCLA. It was February 1, 1984, when he received a call about a donor liver while at his accountant's office. With a limited timeframe to retrieve and successfully transplant the organ, he and two colleagues embarked on a 17-mile journey to St. Joseph Medical Center in Burbank. Before heading to the hospital, they quickly stopped at a 7/11 convenience store to buy a cooler and four bags of ice, as donor organs were transported in an Igloo cooler back then. Dr. Busuttil describes the first human liver transplant he performed as both “intimidating” and “encouraging,” referring to it as the “case of 17” due to the 17-h duration from the donor operation to the transplant's completion, the 17 units of blood required—“which was very, very good,” he says—and the fact that the patient was discharged in 17 days. Dr. Busuttil notes that the patient going home 17 days post-transplant in 1984 was particularly uplifting. This began what would become one of the country's largest and most esteemed transplant centers. Over 7,200 liver transplants would not go without remarkable individual stories. A notable one involves a 1-year-old child diagnosed with a giant hepatic hemangioendothelioma, who underwent a liver transplant on August 8, 1984, his fifth clinical case. Today, she is over 40 years old, happily married and doing well.

Throughout his career, Dr. Busuttil has aimed to reflect a dedication to being a compassionate and considerate physician and surgeon, a commitment to his patients and trainees, and an unwavering focus on saving lives. He emphasizes that liver transplantation embodies this commitment, in addition to vision, self-direction, and the integrity to navigate complex cases. In 2000, Dr. Busuttil remarked, “Burnout does not exist in my vocabulary, I thrive on what I do. Most of these people are at death's door. They are the sickest of the sick. Six months after surgery, you literally cannot recognize them.”

Despite a rigorous clinical workload, Dr. Busuttil has embodied the essence of a surgical scholar, with scientific work encompassing all aspects of liver transplantation, including hepatic physiology, immunology, preservation, technical procedures, and ethical considerations. His laboratory research focuses on organ ischemia-reperfusion injury (IRI), which compromises clinical outcomes and exacerbates the shortage of available donor organs for life-saving surgeries. In the 1970s, he initiated translational studies that defined signaling pathways for pharmacological intervention using steroids to mitigate donor organ IRI. In 1975, he published the results of a randomized, double-blind study in dogs, which documented that pretreatment with methylprednisolone improved heart recovery following ischemia. The study suggested that lysosomal membrane stability and the regulation of cyclic GMP levels might play a crucial role in the mechanism of cardiac ischemic damage (5). Years later, he conducted a randomized single-center double-blind phase 2 clinical trial that revealed how treatment with recombinant P-selectin glycoprotein ligand IgG (rPSGL-Ig) could prevent injury in marginal livers, thereby expanding the organ donor pool (6). This was the first clinical trial to demonstrate a positive impact of an adhesion molecule antagonist on liver IRI in transplant patients.

Dr. Busuttil has been funded by NIH since 1981, with extramural grants exceeding $50M. Throughout his 50-year tenure as a surgical scholar, he has published over 950 peer-reviewed papers, including more than 60 in the *Annals of Surgery* alone, accumulating over 53,000 citations, and achieving an impressive h-index of 114. Additionally, he has written over 90 book chapters and is currently the co-editor of the preeminent textbook, *Transplantation of the Liver*, which is now in its fourth edition.

Dr. Busuttil has served as the President of the International Liver Transplantation Society (ILTS) and the American Society of Transplant Surgeons (ASTS). His numerous accolades reflect contributions to the field, including the ASTS Francis D. Moore Excellence in Mentorship Award, the ASTS Pioneer Award, the Thomas E. Starzl Prize in Surgery and Immunology, the American Surgical Association Medallion for Scientific Achievement, the TTS Award for Education and Training, the ILTS Distinguished Service Award, and the Society of University Surgeons Lifetime Achievement Award, among others. Under Dr. Busuttil's leadership, the ASTS established its Foundation to provide research funding for young transplant surgeon-scientists. The inaugural Ronald & JoAnn Surgeon Scientist Scholarship was presented at the 2024 American Transplant Congress, while honoring Dr. Busuttil's profound impact on generations of transplant surgeons.

Dr. B. has consistently made time for personal interactions with medical students, residents, and fellows, engaging in clinical rounds, one-on-one meetings, and hosting a monthly Journal Club at his home for three decades. His unwavering commitment to education and mentorship has shaped many future leaders in transplantation. Indeed, the UCLA transplant surgery fellowship is internationally acclaimed as one of the premier programs, having trained over 380 transplant surgeons from the United States and other countries. Notably, at least 25 alumni now lead their own transplant programs across the U.S., Asia, and Europe. Some former trainees conveyed to my residence in July 2022 to celebrate Dr. B's retirement (Figure 2). Yes, he achieved remarkable success in his career, but even more significant is his profound impact in nurturing a new generation of leaders in transplantation.

**Figure 2:**
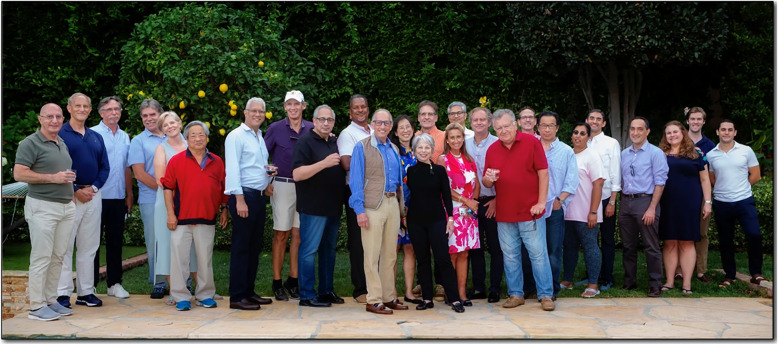
Dr. Busuttil's retirement party (Beverly Hills, CA; July 30, 2022). From left to right: Avi Shaked, John Colonna, Steve Colquhoun, Curtis Holt, Kim Olthoff, David Imagawa, Milan Kinkhabwala, Jim Markmann, Mark Ghobrial, Sherfield Dawson, Ron Busuttil, Pauline Chen, JoAnn Busuttil, Nick Nissen, Sunil Geevarghese, Angeles Baquerizo, Ian Carmody, Jerzy Kupiec-Weglinski, John Duffy, Johnny Hong, Fauzia Butt, Fady Kaldas, Ali Zarrinpar, Keri Lunsford, Daniel O'Brien, Julian Horwitz.

Less known is Ronnie's life beyond the operating room. He is passionate about tennis and running, having completed the NYC marathon twice. His love for cars, likely a trait passed down from his father, a car dealer in Tampa, has led him to attend many renowned races, such as the Monte Carlo Grand Prix and the Indianapolis 500, as well as to compete in the Mille Miglia, a 1,000-mile road race in Italy, three times. Furthermore, I can confirm that the rumors about Dr. B's impressive collection of Italian sports cars sharing his residence address in Bel Air, California, are indeed true. Ronnie often credits his career success to the steadfast support of his wife of over 50 years, JoAnn, along with their two daughters, Amber, and Ashley, and four grandsons, the eldest of whom was aptly named… Oliver.

On a personal note, I am fortunate to have been recruited by Dr. Busuttil from Harvard nearly 30 years ago. His guidance has shaped my life, and I am grateful to call him a friend. Moving from the Brigham and Women's Hospital, the cradle of modern organ transplantation, and leaving Boston for La-La-Land was difficult, but it has proven rewarding. With over four decades of experience in transplant research, I am convinced that fostering mutual understanding and crosstalk between clinical and basic research is crucial for the success of our scientific endeavors. Dr. Busuttil's vision and support have been instrumental in our achievements at the Dumont-UCLA Liver Transplant Laboratory. The continuing NIH funding and publication record in top-tier scientific journals, which extend beyond the transplant field, underscores the broad relevance of our research findings for both the scientific community and clinicians. My initial encounter with Dr. Busuttil at the TTS Congress in Barcelona in 1996 culminated in his well-deserved induction into the pantheon of Medawar Awardees at TTS 2024.

## Data Availability

The original contributions presented in the study are included in the article/Supplementary Material, further inquiries can be directed to the corresponding author.
